# Effects of Black Maca supplement on isokinetics muscular performance of elite women’s handball players: placebo-controlled, crossover study

**DOI:** 10.29219/fnr.v67.10250

**Published:** 2023-12-15

**Authors:** Sunghwun Kang, Byung-O Ahn, Myeong-Hun Park, Seung-Taek Lim, Eunjae Lee

**Affiliations:** 1 Laboratory of Exercise Physiology, College of Art, Culture and Engineering, Kangwon National University, Gangwon-do, Republic of Korea; 2 Interdisciplinary Program in Biohealth-Machinery Convergence Engineering, Kangwon National University, Gangwon-do, Republic of Korea; 3 Hambaek Low Firm, Seoul, Republic of Korea; 4 Republic of Korea Naval Academy, Gyeongsangnam-do, Republic of Korea; 5 Chanmacist, Seoul, Republic of Korea; 6 College of General Education, Kookmin University, Seoul, Republic of Korea; 7 Waseda Institute for Sport Sciences, Waseda University, Saitama, Japan; 8 Institute of Sports and Arts Convergence (ISAC), Inha University, Incheon, Republic of Korea

**Keywords:** black maca, supplementation, inflammation, physical fitness, athletes

## Abstract

**Background:**

The aim of this study was to investigate the changes in isokinetic muscular performance among female adolescent elite handball athletes with the supplementation of Black Maca (BM).

**Methods:**

Eight elite handball athletes were recruited for the present study. The intake capsules contained 2,500 mg of 100% concentrated BM extract and a placebo each, for 4 weeks. Isokinetic muscular performance and physical fitness were measured three times at 4-week intervals after the intake of BM and placebo, including baseline.

**Results:**

The one-way Analysis of Variance (ANOVA) analysis showed a significant improvement in 20 m-shuttle run (*P* < 0.001), 30°/s flexor (*P* < 0.01), and 120°/s flexor (*P* < 0.01) in isokinetic muscle function of the trunk, and 180°/s right extensor (*P* < 0.05), 180°/s left extensor (*P* < 0.05), and 180°/s left flexor (*P* < 0.01) in isokinetic muscle function of the knee, after BM supplementation. Post-hoc analysis indicated that the BM group had significantly higher results compared to the baseline and placebo groups in terms of 20 m-shuttle run, 30°/s flexor and 120°/s flexor of the trunk, 180°/s right extensor, 180°/s left extensor, and 180°/s left flexor of the knee.

**Conclusion:**

BM supplementation can have a positive effect on improving the performance of elite handball players who engage in high-intensity movements by enhancing their isokinetic muscle function and endurance.

## Popular scientific summary

- Black Maca supplementation can have a positive effect on improving the performance of elite handball players.
- Intake of Black Maca supplementation resulted in a significant increase in muscle endurance, isokinetic muscle function of knee and cardiopulmonary endurance.
- Taking Black Maca supplements can help who perform high-intensity movement by reducing muscle contraction fatigue and improving endurance.
- The visible distribution of amino acids and arginine in Black Maca varies depending on the type and content of active ingredients, it may affect anti-stress and antioxidant reactions.

Handball is a highly complex sport that involves several specific movements in both offense and defense, including acceleration, deceleration, turnaround, jumping, passing, throwing, and tackling (1). With unlimited substitutions allowed, players have time to recover off the court, which increases the intensity of in-court movements and allows some players to take on distinct offensive or defensive roles at the elite level (2). Long-term participation in field-based, intermittent, short-distance sports like handball requires high-energy turnover and eccentric muscle action, which cause metabolic and mechanically-induced stress. To overcome these challenges, many athletes use various dietary supplements (3).

The importance of supplements lies in their ability to provide energy, enhance ergogenic potential, improve athletes’ performance, increase power and strength, and enhance body composition, especially during high-intensity, short-duration exercises (4). Elite handball athletes have been shown to exhibit different expression profiles of key genes involved in sports performance-related functions compared to sedentary controls, with gene expression regulation observed after 8 weeks of micronutrient supplementation (5). A significant percentage of handball athletes report using various supplements for fuel during exercise/competition and caffeine-containing supplements to enhance their performance (2). Short-term (5 days) creatine supplementation has been demonstrated to improve maximal lower body strength and fatigue resistance during high-intensity training and/or competition for handball athletes (6). As such, handball athletes often turn to supplements to enhance their performance.

Among various supplements, Maca (*Lepidium meyenii*), a plant that grows in the high altitude of Peru and is used in traditional medicine, has gained attention. Previous studies have reported that administering maca supplements for 21 days increased the swimming time exhaustion of rats and decreased serum lactic acid and liver glycogen concentrations (7). Administering Maca has been shown to strengthen mouse muscle structure, alleviating exercise-induced metabolic stress by upregulating NAD+/NADH, and controlling exercise-induced fatigue in muscles (8). Feeding Maca to mice for 2 weeks improved their anti-fatigue ability, and the higher the concentration of Maca, the better the resistance to fatigue (9). The intake of Maca extracts is expected to have a positive effect on exercise ability, but most previous studies have been conducted on animals.

The safety was demonstrated in 193 adult males and females aged 18–65 years who consumed Maca extract (3,000 mg) for 12 weeks (10). However, most of the previous studies on Maca have been conducted on animals, and only a few have reviewed its effects on humans. Therefore, the aim of this study was to investigate the effects of Black Maca (BM) supplementation on isokinetic muscle performance in female elite handball athletes.

## Materials and methods

### Participation

The participants were eight female elite handball athletes who were recruited for the present study in June 2021. Their physical characteristics were as follows: age = 16.75 ± 0.71 years, height = 168.1 ± 6.23 cm, and weight = 67.61 ± 9.43 kg; mean ± standard deviation [SD].

The study goals and methodology were explained to all subjects who agreed to participate to ensure complete understanding, and the study complied with the ethical standards of the Declaration of Helsinki. All subjects and their parents signed an informed consent form before participation. The Kangwon National University Review Board for Human Subjects approved this study (KWNUIRB-2021-04-013-002).

Table 1 lists the physical characteristics of participants.

**Table 1:** The characteristic of the participants

Variable	Baseline (*n* = 8)	BM (*n* = 8)	Placebo (*n* = 8)
Age (years)	16.75 ± 0.71	16.75 ± 0.71	16.75 ± 0.71
Height (cm)	168.1 ± 6.23	168.7 ± 6.21	168.4 ± 6.38
Weight (kg)	67.61 ± 9.43	67.44 ± 9.92	66.55 ± 9.41
BMI (kg/m^2^)	23.82 ± 2.06	23.57 ± 2.17	23.36 ± 1.98
% fat (%)	24.64 ± 4.38	23.36 ± 5.10	23.43 ± 4.01

Values are expressed as Mean ± SD.

### Procedures

All eight elite handball athletes who participated in this study underwent a physical fitness test and an isokinetics muscular performance test (trunk and knee). The study utilized a randomized, repeated-measured crossover design, where each participant completed two protocols: (1) a 4-week intake of BM, (2) a 4-week intake of placebo, and (3) there was a 2-week wash-out phase between the two supplementation periods. During the first visit to the laboratory, the participants completed the informed consent form, underwent measurements of body composition, physical fitness test, and isokinetics muscular performance test. On the second and third visits after ingestion of BM or placebo, measurements of body composition, physical fitness test, and isokinetics muscular performance test were conducted. The participants visited the laboratory three times during the experiment. All participants were given personal identification numbers to ensure their individual identities could be identified.

### Measurement of body composition

The body composition variables of participants were measured. Weight, height, and percent body fat were measured, using a body composition analyzer (Inbody 720, Body Composition Analyzer; Biospace, Seoul, Korea). Body mass index (BMI) was calculated as weight in kilograms divided by height in meters squared.

### Measurement of physical fitness and isokinetics muscular performance test

Physical fitness and isokinetics muscular performance variables were measured three times: at baseline, after 4 weeks of BM intake, and after 4 weeks of placebo intake. Muscle strength, muscle endurance, flexibility, power, agility, and cardiovascular endurance were evaluated to assess physical fitness and isokinetic muscle function of the trunk and knee.

An isokinetic dynamometer machine (Humac Norm Testing and Rehabilitation, CSMi Medical & Solution, Stoughton, MA, USA) was used to assess muscle strength and endurance of the trunk and knee. Prior to data collection, the isokinetic dynamometer was calibrated according to the manufacturer’s instructions. After calibration, the participants were positioned on the isokinetic dynamometer as per the manufacturer’s recommendations. The participants performed sufficient warm-up exercises, including dynamic stretching for trunk and knee flexion and extension, before the measurements. The maximum isokinetic trunk strength was measured three times at 30°/s, and trunk endurance was measured three times at 120°/s. The range of motion of the trunk during the tests was set from –10° to 70°. The maximum isokinetic knee strength was measured three times at 60°/s, and knee endurance was measured three times at 180°/s. The range of motion of the knee during the tests was set from 0° to 90°.

### Taking BM supplementation and placebo

BM supplement (BLACK MACA 100, Essoco, Kolmar BNH Co., Ltd, Seoul, Korea) was purchased from the Charmacist Pharmacy (Seoul, Korea) on November, 2021. According to the product label, the intake capsules contained 2,500 mg of 100% concentrated BM extract, which included fiber (123.75 mg/2,500 mg), carbohydrate (1,595.5 mg/2,500 mg), protein (192.5 mg/2,500 mg), starch (954.5 mg/2,500 mg), soluble sugar (175.5 mg/2,500 mg), riboflavin (0.019 mg/2,500 mg), potassium (25 mg/2,500 mg), and iron (2.15 mg/2,500 mg). The capsules also contained crystalline cellulose, starch, calcium stearate, and silicon dioxide as fillers. The color of the capsules was dark, and the contents were not visible. Participants were instructed to take one capsule twice a day with water for 4 weeks, without any designated time of day. BM intake was determined using methods described in previous studies (11).

The placebo capsules were identical in appearance, smell, and texture, and were given to participants on the same schedule as the BM capsules. The placebo capsules (gelatin, it was purchased from Charmacist Pharmacy (Seoul, Korea) on November, 2021) contained 2,500 mg of 100% glucose.

Participants were asked to maintain their usual diet for the duration of the study, whether they were taking BM or placebo.

### Statistical analysis

All results are reported as mean ± standard deviation. The data were analyzed using SPSS version 25.0 (SPSS Inc., Chicago, IL, USA). One-way ANOVA was used to assess the differences in time between baseline, post-taking BM supplementation, and placebo (each for 4 weeks). The Bonferroni test was used for post hoc analysis. Statistical significance was accepted at α = 0.05.

## Results

### Change in physical fitness

Table 2 presents the changes in physical fitness among participants.

**Table 2:** Measurements of physical fitness parameters

Variable	Group			*P*	post-hoc
	Baseline (*n* = 8)^a^	BM (*n* = 8)^b^	Placebo (*n* = 8)^c^		
Left grip strength (kg)	27.70 ± 2.29	28.21 ± 1.83	29.03 ± 2.20	0.464	-
Right grip strength (kg)	31.13 ± 4.05	31.58 ± 5.94	32.68 ± 3.72	0.794	-
Sit-ups (rep)	47.38 ± 8.67	50.38 ± 6.35	49.13 ± 6.61	0.718	-
Sit-and-reach (cm)	14.90 ± 5.38	14.93 ± 3.78	14.66 ± 4.10	0.992	-
Long jump (cm)	191.6 ± 7.39	194.8 ± 5.85	193.4 ± 11.4	0.757	-
10 m run (s)	42.75 ± 4.10	45.13 ± 3.44	45.38 ± 5.42	0.434	-
20 m shuttle run (rep)	80.63 ± 8.52	96.88 ± 9.66	92.13 ± 4.55	0.001	b > a, c

Values are expressed as Mean ± SD.

The one-way ANOVA revealed a significant difference in the 20 m-shuttle run (*P* < 0.001). Post-hoc analysis using the Bonferroni test indicated that the 20 m-shuttle run was significantly higher in the BM group compared to the baseline and placebo groups. Grip strength, sit-ups, sit-and-reach, long jump, and 10 m run did not show a significant difference among baseline, BM group, and placebo group.

### Change in isokinetic muscle function of trunk and knee

The changes in isokinetic muscle function of the trunk and knee are shown in Tables 3 and 4.

**Table 3:** Measurements of isokinetic muscle function of trunk parameters

Variable	Group			*P*	post-hoc
	Baseline (*n* = 8)^a^	BM (*n* = 8)^b^	Placebo (*n* = 8)^c^		
30°/s Extensor (%BW)	373.8 ± 80.4	424.6 ± 36.3	358.0 ± 65.9	0.115	-
30°/s Flexor (%BW)	316.4 ± 22.1	329.9 ± 23.0	279.4 ± 31.0	0.002	c < a, b
120°/s Extensor (%BW)	298.8 ± 65.8	326.4 ± 44.5	293.0 ± 27.2	0.356	-
120°/s Flexor (%BW)	374.3 ± 11.7	400.4 ± 22.4	355.6 ± 34.2	0.006	b > c

Values are expressed as Mean ± SD.

**Table 4:** Measurements of isokinetic muscle function of knee parameters

Variable	Group			*P*	post-hoc
	Baseline (*n* = 8)^a^	BM (*n* = 8)^b^	Placebo (*n* = 8)^c^		
60°/s Right Extensor (%BW)	211.0 ± 30.8	220.5 ± 22.0	212.6 ± 39.3	0.814	-
60°/s Left Extensor (%BW)	197.9 ± 22.9	212.9 ± 27.6	199.9 ± 17.1	0.383	-
60°/s Right Flexor (%BW)	109.5 ± 8.8	115.9 ± 12.1	115.3 ± 9.2	0.400	-
60°/s Left Flexor (%BW)	103.8 ± 15.9	116.4 ± 12.6	111.6 ± 13.0	0.211	-
180°/s Right Extensor (%BW)	150.9 ± 11.8	165.1 ± 9.1	152.9 ± 6.2	0.012	b > a, c
180°/s Left Extensor (%BW)	140.4 ± 6.9	154.8 ± 8.8	144.0 ± 12.0	0.017	b > a
180°/s Right Flexor (%BW)	88.4 ± 9.6	97.0 ± 8.3	87.8 ± 7.6	0.076	-
180°/s Left Flexor (%BW)	83.8 ± 8.0	99.6 ± 10.1	90.8 ± 7.2	0.005	b > a

Values are expressed as Mean ± SD.

The one-way ANOVA revealed significant differences in the 30°/s flexor (*P* < 0.01) and 120°/s flexor (*P* < 0.01) in isokinetic muscle function of the trunk. Post-hoc analysis using Bonferroni test indicated that the 30°/s flexor in the BM group was significantly higher and the 120°/s flexor in the BM group was higher. The 30°/s extensor and 120°/s extensor did not show significant differences in the baseline, BM group, and placebo group.

The one-way ANOVA revealed significant differences in the 180°/s right extensor (*P* < 0.05), 180°/s left extensor (*P* < 0.05), and 180°/s left flexor (*P* < 0.01) in isokinetic muscle function of the knee. Post-hoc analysis using Bonferroni test indicated that the 180°/s right extensor, 180°/s left extensor, and 180°/s left flexor in the BM group were significantly higher than in the baseline and placebo group.

## Discussion

In this study, we investigated changes in isokinetic muscular performance after taking BM supplementation or placebo in a crossover study, with each period lasting 4 weeks. The main finding of our study was that intake of BM supplementation resulted in a significant increase in muscle endurance, as indicated by improvements in 180°/s right extensor, 180°/s left extensor, and 180°/s left flexor in isokinetic muscle function of knee. Furthermore, we observed improvements in cardiopulmonary endurance, as indicated by an increase in the 20 m-shuttle run performance of elite handball athletes who took BM supplementation.

Isokinetic muscle function is considered a more useful parameter than muscle strength when referring to muscle performance, as it characterizes muscle function according to the type of isometric muscular function (12). Muscle strength has traditionally been expressed as the strength relative to total body weight, which is considered a measure of muscle quality (13). The evaluation of muscle strength, agility, and athletes’ performance is often described in terms of isokinetic knee muscle strength and functional association of the quadriceps and hamstring muscles (14). Additionally, trunk flexion and extension strength is greater in high-level athletes than in non-athletes and may be associated with athletic performance (15). For this reason, this study measured isokinetic muscle function of the knee and trunk muscles, and the results showed that 30°/s flexor and 120°/s flexor in isokinetic muscle function of the trunk and 180°/s right extensor, 180°/s left extensor, and 180°/s left flexor in isokinetic muscle function of the knee were significantly higher after taking BM supplementation. Although the visible distribution of Maca’s amino acids, amide alkaloids, imidazolium alkaloids, and saccharides varies depending on the types and contents of active ingredients such as glucosinolate, essential oil, macamid, and macaene, Maca is reported to have an effect on reproductive function, anti-stress response, anti-osteoporosis, and anti-tumor activity (16). In particular, BM supplementation shows excellent efficacy in anti-stress response and typically shows an excellent response to antioxidants. Zhu et al. reported that Maca inhibited the reduction in viability and accumulation of reactive oxygen species (ROS) by treatment with H2O2 in C2C12 skeletal muscle cells (8). ROS decomposes biological substances such as deoxyribonucleic acid, lipids, and proteins, which might lead to oxidative tissue damage (17). Exercise-induced ROS is required to produce natural muscle strength, but high levels of ROS concentration appear to cause contractile dysfunction (18). Handball athletes require proper management due to their high intensity of training and competition, and increased ROS affects muscle fatigue, which adversely affects their performance (19). In this study, all variables of constant isokinetic muscle function of the trunk and knee tended to increase after BM intake. In particular, we found a significant improvement in 180°/s right extensor and 180°/s left extensor in isokinetic muscle function of the knee after BM intake. Xaverova et al. reported that the bilateral force deficit for knee concentric extension of handball athletes was significantly higher in the women’s junior team compared to the women’s national team (20), requiring muscle function improvement to reduce the risk of injury, as bilateral force deficit occurred more frequently in the women’s national team. BM supplementation might help female handball athletes improve their muscle strength to prevent such injuries.

In handball, maintaining a high level of endurance is important to sustain a high level of play throughout the entire game (2 × 30 min) (21). Competition-based training that includes specific movements should be preferred over shuttle runs, high-intensity running training, or repetitive sprint training to improve general or specific coordination in team handball (22). This study shows that the 20 m-shuttle run was significantly higher in the BM group than the baseline and placebo group. The 20 m-shuttle run is typically used to measure cardiopulmonary endurance, which can be replaced by VO2max as a field test (23). Maca administration also increased the relative proportion of contraction and relaxation before ischemia/recurrent, suggesting that the heart was better prepared for ischemia challenges with higher energy storage and muscle economy (24). Arginine, which is present in Maca, affects endurance and heart (25). Rezaei et al. reported that meta-analysis indicated that supplementation with arginine could increase VO2 max in healthy people (26). Speer et al. also support the fact that the combination of arginine and citrulline might increase nitric oxide production and enhance vasodilation, thereby improving athletes’ performance (27). Increased bioavailability of nitric oxide might induce physiological effects that improve endurance exercise performance (28). Furthermore, as mentioned earlier, BM intake has a positive effect on anti-oxidants, which can improve the 20 m-shuttle run for elite handball athletes. Maca supplements also play a protective role in cyclophosphamide-induced hepatotoxicity, where the Keap1-Nrf2 signaling pathway is regulated to improve oxidative stress, energy metabolism, and restore mitochondrial respiration to prevent liver damage (29). Therefore, for athletes with high-intensity training and competition, including handball, BM intake can have a positive effect on improving their performance by enhancing their cardiopulmonary endurance.

The present study had some limitations. The sample size was small, which limited our ability to determine the significance of the results. Future studies with larger sample sizes and a control group are required to determine the effectiveness of BM supplementation on changes in isokinetic muscle function and physical fitness in athletes. Another limitation was that we did not analyze ROS directly. Although BM has a significant effect on ROS reduction, future studies will require an analysis of ROS. And the study period was short; therefore, more longitudinal studies are required to investigate the effects of BM supplementation. Finally, we assumed that there were no significant differences in physical activity amount and diet because the participants were lived in in the same dormitory and living in groups during the study. However, physical activity and diet kcal factors should be considered in future studies.

## Conclusion

In conclusion, this study suggests that intake of BM supplementation can change isokinetic muscle function and improve cardiopulmonary endurance. Although the visible distribution of amino acids and arginine in BM varies depending on the type and content of active ingredients, it may affect anti-stress and antioxidant reactions. Oxidants are probably detected at low levels during rest and at high levels during muscle contraction, and they contribute to muscle fatigue because the loss of function can be delayed by ROS-specific antioxidants (19). Therefore, intake of BM supplementation can have a positive effect on the improvement of elite handball players’ performance, who perform high-intensity movements by reducing muscle contraction fatigue and improving endurance.

## Data Availability

The data will be provided upon reasonable request.
